# The Impact of the Carer Support Needs Assessment Tool (CSNAT) in Community Palliative Care Using a Stepped Wedge Cluster Trial

**DOI:** 10.1371/journal.pone.0123012

**Published:** 2015-04-07

**Authors:** Samar M. Aoun, Gunn Grande, Denise Howting, Kathleen Deas, Chris Toye, Lakkhina Troeung, Kelli Stajduhar, Gail Ewing

**Affiliations:** 1 School of Nursing and Midwifery, Curtin University, Perth, Western Australia, Australia; 2 School of Nursing, Midwifery & Social Work, The University of Manchester, Manchester, United Kingdom; 3 School of Population Health, The University of Western Australia, Crawley, Western Australia, Australia; 4 School of Medicine, The University of Notre Dame Australia, Fremantle, Western Australia, Australia; 5 School of Nursing and Centre on Aging, University of Victoria, Victoria, British Columbia, Canada; 6 Centre for Family Research, University of Cambridge, Cambridge, United Kingdom; University of Geneva, SWITZERLAND

## Abstract

Family caregiving towards the end-of-life entails considerable emotional, social, financial and physical costs for caregivers. Evidence suggests that good support can improve caregiver psychological outcomes. The primary aim of this study was to investigate the impact of using the carer support needs assessment tool (CSNAT), as an intervention to identify and address support needs in end of life home care, on family caregiver outcomes. A stepped wedge design was used to trial the CSNAT intervention in three bases of Silver Chain Hospice Care in Western Australia, 2012-14. The intervention consisted of at least two visits from nurses (2-3 weeks apart) to identify, review and address caregivers’ needs. The outcome measures for the intervention and control groups were caregiver strain and distress as measured by the Family Appraisal of Caregiving Questionnaire (FACQ-PC), caregiver mental and physical health as measured by SF-12v2, and caregiver workload as measured by extent of caregiver assistance with activities of daily living, at baseline and follow up. Total recruitment was 620. There was 45% attrition for each group between baseline and follow-up mainly due to patient deaths resulting in 322 caregivers completing the study (233 in the intervention group and 89 in the control group). At follow-up, the intervention group showed significant reduction in caregiver strain relative to controls, p=0.018, *d*=0.348 (95% CI 0.25 to 0.41). Priority support needs identified by caregivers included knowing what to expect in the future, having time for yourself in the day and dealing with your feelings and worries. Despite the challenges at the clinician, organisational and trial levels, the CSNAT intervention led to an improvement in caregiver strain. Effective implementation of an evidence-informed and caregiver-led tool represents a necessary step towards helping palliative care providers better assess and address caregiver needs, ensuring adequate family caregiver support and reduction in caregiver strain.

## Introduction

Family caregivers providing informal care in the home are major sources of support for people with long-term illnesses and severe disabilities. Despite the significant contribution of family caregivers to the economy in many developed countries, there is considerable evidence in the national and international literature, of the physical, psychological and social morbidity associated with caregiving, highlighted in studies with adverse titles, such as: “Caring enough to be poor” [1]; “Ignored and invisible” [2]; “Caregiving as a risk factor for mortality” [3]; “Missed Opportunities” [4]; “Warning—caring is a health hazard” [5]; “Family caregivers and leisure: an oxymoron?” [6]; “The hardest thing we have ever done: The social impact of caring for a dying person in Australia” [7]. Caregivers tend to be overlooked and often referred to as “hidden patients” [8].

Studies have shown that caregivers with better experiences of end-of-life care were substantially less depressed over time and had better bereavement outcomes [9,10]. While a wide research literature base exists on caregiver interventions [11–13], more needs to be achieved regarding demonstrating and validating robust and effective caregiver support interventions and establishing ways of disseminating and sharing evidence of successful interventions that address caregiver experiences and needs [14], and ameliorate the effects of caregiving [15,16].

As family caregivers experience numerous support needs, the challenge is to find accessible and acceptable approaches for caregivers in order to meet their needs or interventions that address the range of needs [17]. While ‘one to one’ interventions for caregivers have been reported to be useful though expensive, group interventions can be hard for many caregivers to undertake because of the need to be away from the care recipient [18,19].

Family caregivers need to be supported in their central role of caring and their support needs well identified by the service providers. However, brief practical tools to assess their support needs have been lacking or consisted of indirect measures of caregiving difficulties, feelings of burden, meaning and relationship issues [20]. The Carer Support Needs Assessment Tool (CSNAT) is a validated evidence based tool developed for home based care to fill the gap between validated research tools that have minor relevance to actual practice and more ad hoc service-based assessment forms that lack an adequate evidence base [21,22].

The CSNAT adopts a screening format, structured around 14 broad support domains. This format allows it to be brief but also comprehensive, enabling caregivers to identify the domains in which they require further support which can then be discussed with health professionals. Each item represents a core family caregiver support domain in end of life home care and these domains fall into two distinct groupings: those that enable the caregiver to care and those that enable more direct support for themselves.

### Aims

Aims of the study were to investigate the impact of using the carer support needs assessment tool (CSNAT), as an intervention to identify and address support needs in end of life home care, on family caregiver outcomes such as strain, distress and mental and physical health; and to describe the implementation strategies, identified support needs and provided solutions to address the needs of family caregivers.

## Method

The study was approved by the Curtin University Human Research Ethics Committee (HR 24/2011) and the Silver Chain Human Research Ethics Committee (EC App 068). All participants provided written informed consent to participate in this study and the two ethics committees approved this consent procedure. All aspects of the trial conformed to CONSORT requirements.

### Setting

The trial was conducted in Perth, Western Australia, in three sites of the Silver Chain Hospice Care Service (SCHCS) in 2012–14. Silver Chain is Australia’s largest provider of home based palliative care. The service is provided by an interdisciplinary team comprising general practitioners with a special interest in palliative care, medical consultants, registrars, resident medical officers, palliative care specialist nurses, counsellors, chaplains, care assistants, social workers and volunteers, who work with the patient to control symptoms and address psychosocial needs. Typically, nurses visit patients weekly and increase visits according to client needs for care and symptom management and care assistants provide personal care visits three times per week to daily depending on patient needs. The average length of stay with the service is approximately 90 days.

### Trial design

**Fig 1:**
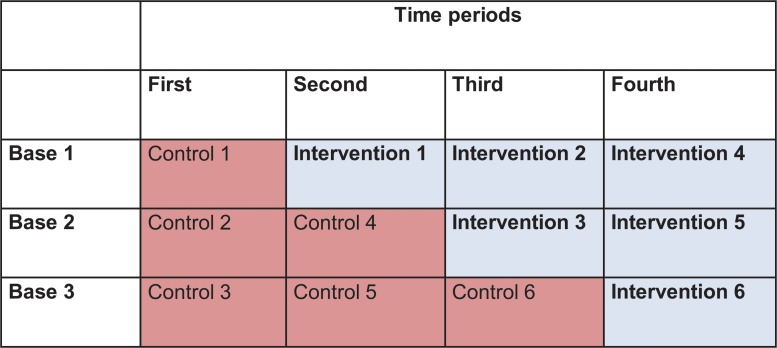
Stepped Wedge Trial Design.

The study was a stepped-wedge cluster non-randomised trial. Each of the three bases undertook the intervention in a stepped sequence wherein the CSNAT was introduced at each site at different time points, with the pre-intervention period serving as a control period (Fig 1). Intervention start times for each base were assigned by the researcher. The sequence was not randomised because the service preferred to start with the site that undertook the pilot study (described below), being more ready to implement the intervention. This also gave more time for the other two sites to settle into this research study, in terms of adapting to all aspects of the research process, before they were ready to implement the intervention.

A stepped-wedge design was considered more appropriate, cost effective and practical than a cluster randomised controlled trial. As all sites start as controls, any baseline differences between sites can be ascertained. Also, a randomized controlled trial within each base was considered not feasible because contamination could occur as staff can be visiting clients in both groups within each base [23,24].

### Participants

Participants were primary family caregivers of terminally ill patients (with cancer or non-cancer diagnoses) referred to Silver Chain Hospice Care. A primary family caregiver was defined as a person who, without payment, provided physical care (and emotional care) to a person who was expected to die during provision of the caring role. This care could be provided on a daily or intermittent basis. All adult caregivers (aged 18 years or older) who were able to read and write in English were eligible for the study, unless the service had concerns about their ability to cope with research because of the exceptionally high levels of distress. Family caregivers with a known cognitive impairment were excluded based on the nurses’ clinical judgment.

### Intervention and Training

The CSNAT intervention consisted of at least two visits from nurses to caregivers, 2 to 3 weeks apart, where nurses incorporated the CSNAT into a practitioner facilitated but caregiver-led approach to needs assessment and support. Family caregivers identified domains where they needed more support. This was accomplished by the CSNAT being either self-completed by the family caregiver or completed jointly with the nurse. Then a conversation took place to determine individual needs and the caregiver’s priorities were discussed with the nurse to agree on actions/solutions and a shared action plan.

Therefore nurses were trained in the use of the CSNAT as a caregiver-led approach for identifying and addressing caregiver needs rather than the traditional way of professional-led approach. One training session of 2–3 hours long was provided (mostly in groups of 6–8) at the start of data collection for the intervention. Therefore, a total of six main training sessions (2 sessions for each site) were undertaken to accommodate the availability of staff. Due to the additional time pressure training sessions were perceived to add to the nurses’ busy schedules, we were asked to keep sessions short and not so frequent. These initial training sessions were followed towards the middle of the data collection period by three refresher sessions (1 session for each site) lasting 1–2 hours to discuss nurses’ experiences, issues with recruiting and completing the intervention. In addition, the research team had monthly meetings with the designated champion of the project in the service (a senior clinical nurse manager) to follow progress and discuss challenges in each site. Suggestions from the research team were relayed back by the champion to the recruiting nurses during their regular weekly clinical meetings. Furthermore, nurses had daily access by phone to the advice of the research nurse, and texting was the preferred way for individual nurses to liaise with the research nurse.

All three sites commenced as control sites for the first time period (Fig 1). As each site entered the intervention phase, the training for each intervention site was timed to be provided immediately prior to the intervention commencing at the particular site. In general, each site had its own staff, but sometimes a few were called upon to assist in another site for a short period if there were staff shortages due to sickness or other operational matters. However, to avoid contamination across sites, efforts were made to ensure the nurses with intervention training were not available to work with control group participants at another site.

The control group received ‘standard practice’ which consisted of the staff meeting with the caregiver during the client visit and discussing caregiver needs on an informal basis. Following this discussion, the staff member would offer services and equipment that Silver Chain was able to provide. However many conversations were on an ad hoc basis and not documented.

### Recruitment and data collection

At the start of the study, family caregivers of clients newly admitted or already receiving palliative care were invited by participating nurses to take part at the first feasible opportunity during a face to face visit. Nurses obtained written informed consent for the study and for the researcher to contact them by phone to undertake the pre and post intervention outcome measures. For the intervention group, once the researcher completed the pre-intervention outcome measures by phone with the caregiver, she informed the nurse who completed at least two visits implementing the CSNAT intervention. Once the nurse completed the CSNAT visits, the researcher was informed so post intervention outcome measures could be collected from the caregiver by phone, aiming to do it within three to seven days. However this depended on how soon the nurse informed the researcher and also on caregivers’ availability. To ensure that this research process worked well, liaison between the researcher and the nurses occurred almost on a daily basis. Deaths were monitored throughout the data collection period to ensure that bereaved carers were not contacted by phone for completing the follow-up measures, as it was not sensitive to do so once patient death occurred.

Nurses were also asked to keep a record on a specially designed form of those caregivers who did not participate in the study (control or intervention, gender, age and reason for non- participation).

### Outcomes

Data collection by the researcher was undertaken at the individual participant level and conducted at pre and post intervention over the phone for the two groups. Outcome measures (Caregiver Survey) were collected by the researcher at both time points. Demographic data were collected at baseline only and consisted of:

- Caregivers: age, gender, marital status, education, employment status, living arrangements, ethnicity, relationship to patient; other caregiving responsibilities.
- Patients: age, gender, type of diagnosis, length of diagnosis, period receiving palliative care.

#### Primary outcome

The primary outcome was caregiver strain and distress as measured by the 2 subscales of the Family Appraisal of Caregiving Questionnaire (FACQ-PC) where strain has 8 items and distress has 4 items [25]. The appraisal of the strain subscale (also referred to as index of burden) includes “items that have been closely linked to outcome, namely, role overload (the extent to which caregivers perceive that they are physically or emotionally depleted by their caregiving activities), and role captivity (the extent to which caregivers feel trapped by their responsibilities and isolated)” ([25], p. 615). The distress subscale represents “negative emotional responses associated with caregiving such as anxiety and depression as well as feelings of guilt in the caregiver-care recipient relationship” ([25], p. 616). Psychometric analyses demonstrate good construct validity. Internal reliability estimates range from 0.75–0.86. Scores range from 5 = strongly agree to 1 = strongly disagree.

#### Secondary outcomes

Secondary outcomes were caregiver mental and physical wellbeing as measured by SF-12v2 and caregiver workload as measured by caregiver assistance with Activities of Daily Living.

The SF-12v2 consists of 12 questions; relating to: physical health problems, bodily pain, general health perceptions, vitality (energy/fatigue), social functioning, role limitations and general mental health (psychological distress and psychological well-being). Reliability estimates range from 0.93 to 0.95 [26].

Caregiver workload was measured by the nature and extent of assistance provided by the family caregiver with a range of Activities of Daily Living (ADLs, such as feeding and toileting) and Instrumental Activities of Daily Living (IADLs such as transport and shopping). Scores are: 4 = assistance all of the time; 3 = assistance most of the time; 2 = occasional assistance; 1 = no assistance required.

### Pilot study

The study was first pilot tested to assess the feasibility and acceptability of the tools and the data collection process by both nurses and caregivers, in one of the three service bases. Five nurses and 21 family caregivers participated in the pilot study for a period of four months in 2011 and gave feedback that was incorporated in the present study. In general, completing the CSNAT was very acceptable to the nurses and caregivers. The Caregiver Survey, which incorporated the outcome measures, was also acceptable to the caregivers in terms of content and length. Recommended changes that were implemented in the present study included:

- CSNAT visits need to be undertaken by nurses every 2 weeks rather than 4 weeks to minimise attrition due to patient deaths
- Nurses reported that it was time consuming for them during their visit to explain and assist caregivers to complete the Caregiver Survey. Therefore, the data collection method needed to change from caregivers self-administering the Caregiver Survey and then posting it to the research team. Instead the research officer would assist caregivers to complete the survey via a telephone call at a time that was convenient to them pre and post intervention, thus reducing the burden of completing it during the nurse’s visit.
- The service decided to have more nurses involved in the larger trial to take the pressure off recruiting, especially when the service encounters the same challenges experienced in the pilot study such as higher patient workload or staff shortages in a particular time period.

### Sample size

Previous research has found that caregivers who felt psychologically unsupported during caregiving report worse mental health post bereavement (effect size 0.67) [27]. Assuming a more modest effect size of 0.41 for the primary outcome measure (FACQ-PC), a sample size of 95 in each group (intervention and control) would give 80% power to demonstrate an effect of the intervention at alpha <0.05, two-tailed test between two independent groups using a standard RCT [28]. A design effect correction of 1.62 was then applied to the estimated sample size to adjust for the cluster design of the trial based on a conservative intraclass correlation coefficient (ICC) of 0.01 and average cluster size of 63, The resulting required sample was 308 (154 caregivers per group). We also applied a correction for an estimated 30% attrition rate, resulting in a final a priori sample size of 440, or 220 in each group. This sample size was considered feasible based on the fact that on average 200 new patient referrals are received per month.

### Statistical analysis

All statistical analyses were conducted using SPSS 22. Statistical significance was determined at an alpha value of 0.05. Analyses of this trial were on a per protocol basis.

Continuous variables were reported as mean ± standard deviation and categorical variables are reported as n (%). Baseline differences between Intervention and Control groups were assessed using Mann-Whitney U Tests for continuous variables and Chi-square tests or Fisher’s Exact Test (where cell sizes were less 5) for categorical variables. Cohen’s *d* and 95% Confidence Intervals (CI) for calculating effect sizes was determined for the outcome measures.

Generalized linear mixed modelling (GLMM) was used to examine the efficacy of the CSNAT intervention from baseline to post-treatment. GLMM is a regression-based approach that provides a more powerful means of analysing clustered data when compared with more conventional procedures for the comparison of group means by explicitly accounting for intra-cluster correlation [29]. Moreover, GLMM is robust to unequal group sizes, which was a significant issue in the present study [30].

A series of six GLMMs were run, one for each outcome. Each model included two random effects; Base (to account for the intra-class correlation within bases) and Time (to account for the correlation between repeated observations of the same individual). Fixed effects included were; Time, Base, Condition, the interaction between Time and Condition (Time x Condition), and the interaction between Time and Base (Time x Base). The Time × Condition interaction effect was the primary variable of interest in each model with a significant interaction effect indicating a differential rate of change in outcomes between the intervention and control conditions from baseline to post-treatment. Five covariates were also included in each GLMM to adjust for significant baseline differences between groups; Age of caregiver, effect of caring on work, diagnosis of patient, length of palliative care, and length of Silver Chain admission.

## Results

### Participant flow

**Fig 2:**
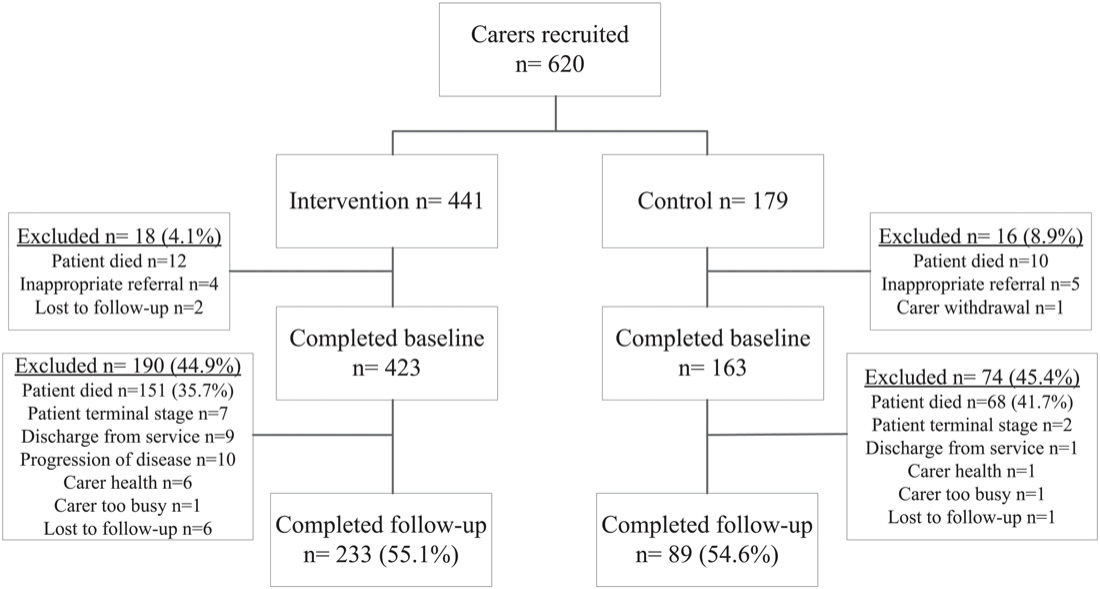
Flowchart of participants in the trial.

Fig 2 displays the participant flow for the study. In total, 620 caregivers (Intervention n = 441, Control n = 179) were recruited and/or referred over a time period of 24 months (March 2012 to February 2014). The observed group imbalance was due to slow recruitment at the start of the study as a result of logistical and operational service constraints, when all sites were recruiting for the control group.

Thirty four individuals withdrew prior to baseline measures. Reasons for discontinuation included; patient death (n = 22), did not meet eligibility criteria (n = 9), lost to follow-up (n = 2) or caregiver withdrawal (n = 1). The remaining 586 caregivers (Intervention n = 423, Control n = 163) completed baseline measures. Two hundred and thirty three participants (55.1%) completed the study in the intervention group and 89 (54.6%) in the control group after baseline measures. The attrition rate between baseline and follow-up was similar for both groups (44.9% for the intervention group and 45.4% for the control group). This rate was significantly higher than our estimated rate of 30%. Attrition was predominantly due to patient deaths in both groups (Intervention: n = 151, 79.4% of all those withdrawn, Control: n = 68, 91.9% of all those withdrawn). Our analyses were based on the study completers sample only (n = 322), or per protocol.

Compared to drop-outs, participants who completed the study had a longer period of caring, were less likely to be in paid employment, and had more from a non-English background. All other demographic variables were equivalent and patient profiles did not differ.

### Participant characteristics

Table 1 displays demographic data at baseline for family caregivers who completed the study in the two groups. Caregivers did not differ between the two groups on most characteristics except that the intervention participants were slightly younger than the control participants, currently engaged in paid employment, had care recipients with cancer diagnoses, with shorter median lengths of stay (LOS) with Silver Chain and shorter median LOS in palliative care.

**Table 1:** Characteristics of groups who completed the CSNAT study.

		Study Group		
		Intervention N = 233	Control N = 89	*p*-value
**Silver Chain Base**	Base 1	108 (46.6%)	15 (16.9%)	
	Base 2	65 (27.9%)	35 (39.3%)	
	Base 3	60 (25.8%)	39 (43.8%)	
**FAMILY CARER**				
Gender				0.094
	Male	69 (29.6%)	18 (20.2%)	
	Female	164 (70.4%)	71 (79.8%)	
Age (yrs.)				0.016
	Mean ± SD	62.1 ± 12.4	65.5 ± 13.16	
	Median (Range min., max.)	62.0 (20, 88)	67.0 (33, 92)	
Marital status				0.217
	Never married	13 (5.6%)	2 (2.2%)	
	Widowed	7 (3.0%)	2 (2.2%)	
	Divorced/separated	11 (4.7%)	9 (10.1%)	
	Married/de facto	202 (86.7%)	76 (85.4%)	
Cultural background				0.111
	Australian	129 (55.4%)	60 (67.4%)	
	Other English speaking	66 (28.3%)	21 (23.6%)	
	Non-English speaking	38 (16.3%)	8 (9.0%)	
Usual employment				0.144
	Paid employment	74 (31.8%)	17 (19.1%)	
	Pensioner	96 (41.2%)	44 (49.4%)	
	Self-funded retiree	35 (15.0%)	15 (16.9%)	
	Other	28 (12.0%)	13 (14.6%)	
Education				0.502
	No formal education	1 (0.4%)	0	
	Primary	5 (2.1%)	1 (1.1%)	
	Secondary	133 (57.1%)	57 (64.0%)	
	Tertiary	94 (40.3%)	31 (34.8%)	
Living arrangements				0.158
	Private residence	223 (95.7%)	81 (91.0%)	
	Retirement village	7 (3.0%)	7 (7.9%)	
	Other	3 (1.3%)	1 (1.1%)	
Relationship				0.644
	Spouse	157 (67.4%)	63 (70.8%)	
	Parent	4 (1.7%)	3 (3.4%)	
	Adult Child	52 (22.3%)	16 (18.0%)	
	Sibling	5 (2.1%)	3 (3.4%)	
	Other	15 (6.4%)	4 (4.5%)	
Caring affected work				0.007
	Gave up job	41 (17.7%)	16 (18.0%)	
	Reduced hours	27 (11.6%)	8 (9.0%)	
	No change	22 (9.5%)	2 (2.2%)	
	Not working	115 (49.6%)	60 (67.4%)	
	Other	27 (11.6%)	3 (3.4%)	
Other caring responsibilities				0.375
	Yes	57 (24.5%)	17 (19.1%)	
	No	176 (75.5%)	72 (80.9%)	
Caring length (months)	Mean (± SD)	21.7 ± 43.48	18.4 ± 24.39	
	Median (Range)	10.0 (0.3, 420)	11.0 (1, 144)	0.498
**PATIENT**				
Patient gender				0.452
	Male	130 (55.8%)	54 (60.7%)	
	Female	103 (44.2%)	35 (39.3%)	
Patient age (years)				0.172
	Mean (± SD)	70.3 ± 13.37	72.1 ± 14.29	
	Median (Range)	72.0 (28, 94)	74.0 (4, 93)	
Diagnosis				0.029
	Cancer	175 (75.1%)	66 (74.2%)	
	Cancer + non-cancer	39 (16.7%)	8 (9.0%)	
	Non-cancer	19 (8.2%)	15 (16.9%)	
Length of diagnosis (months)	Mean (± SD)	30.3 ± 49.84	31.1 ± 50.89	
	Median (Range)	13.0 (0.3, 420)	13.0 (1, 400)	0.697
Length of palliative care (months)	Mean (± SD)	2.9 ± 4.24	6.0 ± 8.29	
	Median (Range)	1.5 (0.3, 29)	4.0 (0.3, 72)	0.000
Length of stay with Silver Chain (months)	Mean (± SD)	2.3 ± 3.75	5.1 ± 5.19	
	Median (Range)	0.9(0.03, 4.16)	3.2(0.03, 7.95)	0.000

Information was collected on 121 non-participants and their basic demographics did not differ from those of participants: 71% were women, mean age was 65 years and 75% were in the intervention group. The predominant reasons for not participating were: too busy/overwhelmed (43%), not interested (13%) and patient deteriorating or at terminal stage (8%).

### Primary outcome: FACQ-PC

The intervention was associated with a significant reduction in Caregiver Strain, while control participants experienced an increase in strain over the study period (Table 2). Mean reduction in caregiver strain was 0.08 for intervention participants compared with an increase of 0.09 for the control group after adjusting for covariates, p = 0.018, *d* = 0.348 (95% CI 0.25 to 0.41). Similarly, intervention participants experienced a mean reduction of 0.10 in Caregiver Distress scores from baseline to follow-up, while control participants experienced a mean increase of 0.04, although this result was not statistically significant after adjusting for covariates, p = 0.26, *d* = 0.23 (95%CI 0.11 to 0.30).

**Table 2:** Mean change in domain scores for FACQ-PC, SF-12v2, and ADL/IADL from baseline to follow-up.

		Time	Intervention (n = 233)	Control (n = 89)	Adjusted *p*-value^1^	Effect size^2^ (95%CI)
			Mean (SD)	Mean (SD)		
**FACQ-PC** ^**3**^	Caregiver Strain	1	2.92 (0.87)	2.92 (0.87)	0.018*	0.348 (0.25–0.41)
		2	2.84 (0.72)	3.01 (0.82)		
	Caregiver Distress	1	3.13 (0.76)	3.20 (0.88)	0.261	0.231 (0.11–0.30)
		2	3.03 (0.70)	3.24 (0.84)		
**SF-12v2** ^**4**^	MCS	1	43.13 (10.59)	44.90 (12.56)	0.678	0.156 (-1.70–1.40)
		2	44.48 (10.51)	44.85 (11.53)		
	PCS	1	51.93 (9.65)	50.53 (11.78)	0.975	0.000 (-1.36–0.87)
		2	51.29 (9.97)	48.94 (11.12)		
**Assistance with ADL’s** ^**3**^	ADL	1	1.72 (0.76)	1.71 (0.80)	0.090	0.255 (0.13–0.34)
		2	1.85 (0.82)	2.00 (0.88)		
	IADL	1	3.46 (0.49)	3.43 (0.57)	0.502	0.133 (0.04–0.17)
		2	3.47 (0.50)	3.48 (0.56)		

^1^ p-value for the Time x Condition interaction effect adjusted for cluster effect, age of carer, effect of caring on work, diagnosis of patient, length of palliative care, length of Silver Chain admission

^2^ Cohen’s *d*

^3^ Higher scores indicate greater strain, stress and tasks assisting the patient

^4^ Higher scores indicate greater wellness.

* *p* < 0.01

### Secondary outcomes

The intervention was also associated with an improvement in the SF12v2 Mental Component Score (MCS). Mean improvement in MCS was 1.42 for the intervention group compared with a decrease of 0.05 for the control group, although this result was not statistically significant after adjusting for covariates, p = 0.67, *d* = 0.16 (95%CI -1.70 to 1.40). Both groups showed a worsening on the SF12v2 Physical Component Score (PCS) over the two time points, but these differences were not significant (Table 2).

Both groups showed increased caregiver workload assisting with patient activities of daily living (ADLs) from baseline. However, this increase was smaller in the intervention group for both the ADL and IADL measures, though the difference between groups was not statistically significant after controlling for covariates.

### Implementation of the CSNAT

Two hundred and thirty three family caregivers experienced the CSNAT intervention. The mean period between the two visits by nurses, where CSNAT was undertaken, was 23 days (SD = 14), and the mean period between baseline and follow-up data collection for outcome measures was 43 days (SD = 20).

#### Family caregivers’ support needs

**Fig 3:**
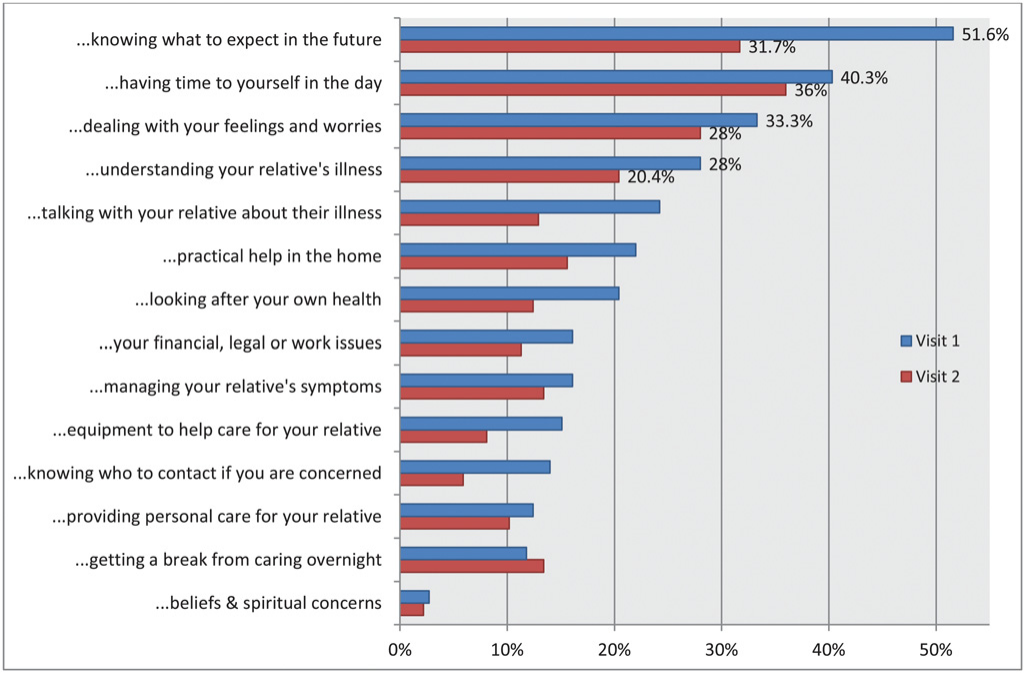
Percentage of family carers expressing need for more support with each CSNAT domain during first and second visit from nurse.

The top four support needs reported by family caregivers at the first and second nurse visits consisted of (Fig 3): knowing what to expect in the future (52%, 32% respectively), having time for yourself in the day (40%, 36% respectively), dealing with your feelings and worries (33%, 28% respectively) and understanding your relative’s illness (28%, 20% respectively).

#### Solutions provided

The solutions put in place by the nurses for “knowing what to expect in the future” consisted of discussions (using nurses’ quotes) “on what to expect as [client’s] condition deteriorates and death nears”, “recommended the Helpful Comfort Measures booklet” or “long talk about end of life care/dying at home/ symptom management with [infusion] pump/ extra input from care aides”. For the second priority on “having time for yourself in the day”, the nurses discussed "time out", the options of respite care, family rosters, volunteers and personal alarm for the client.

The solutions put in place for the third priority “dealing with your feelings and worries” consisted of: seeing a counsellor, a chaplain, encouragement to attend the day centre at Community Hospice and to call on friends for support, and referral to social worker regarding financial aspects of funerals. For the fourth priority “understanding your relative’s illness”, the nurses provided explanations on matters such as: signs of seizure activity and how to respond, more invasive tests might be required so need to gain more information from the specialist and providing information for the children from the cancer support service.

## Discussion

This study is the first to test the CSNAT intervention through the caregiving period (in contrast to an UK trial that focused on post bereavement outcome measures [31]), using a control group, and all sites received the intervention using the stepped wedge design. The Intervention was associated with a significant reduction in caregiver strain. A reduction in caregiver distress was also indicated but not significant after controlling for covariates. Both groups showed an increase in caregiver workload (assisting with ADLs) which was expected given the nature of disease progression with time. However the intervention was associated with a smaller increase in caregiver workload. The differences in SF12v2 scores were not significant maybe due to SF12v2 not accurately capturing the outcome for the study within a short time period of the intervention or the intervention had limited effect in this case.

The observed significant small to moderate effect size of this intervention (d = 0.348, 95% CI 0.25 to 0.41) is comparable if not larger than those reported in a meta-analysis of caregiver interventions by Northouse et al [32]. Specifically, reported interventions on appraisal of caregiver burden had effect sizes (Hedges’ *g*) with confidence intervals ranging from 0.08 (95% CI -0.19 to 0.34) to 0.22 (95% CI 0.08 to 0.35). Our obtained effect size of 0.348 means that 64% of the control group would have a score below the average participant in the intervention group [33]. Northouse et al concluded that even though effects were small to moderate in size, these interventions have significantly reduced caregivers’ burden among other positive outcomes, and therefore they show promise of achieving clinically significant outcomes, “as these interventions produce more prepared, less distressed caregivers which in turn is likely to result in more positive benefits for patients” ([32], p. 12).

The profile of the study sample is similar to that in many studies where family caregivers are predominantly women, spouses, retirees, and caring for older family members with mainly a malignant disease, and in particular comparable to a study from the UK [21]. It is also worth noting that the four priorities in support needs for family caregivers in this Australian study were similar to those obtained in the UK study [21].

Most of the solutions put in place had the opportunity to be enacted upon in the period between baseline and follow-up (mean period was 43 days), as with close to half of the patients dying, it was not feasible to extend further the timing of obtaining the follow-up outcome measures. By structuring and reviewing caregivers’ needs 2–3 weeks apart, Fig 3 gives some demonstration of a steady reduction in the caregivers’ perception of needs between the two CSNAT visits, thus providing good evidence that some of the needs have been addressed through the solutions provided. This reinforces the benefit of systematically repeating this review of needs using the CSNAT approach. The single domain that revealed a rise in need over time was getting a break overnight, which became important across time. This could reflect the benefit of recognition by caregivers that their own needs have become more visible and acknowledged now, thus feeling that their request to have a break is more legitimised [21,34].

We believe the intervention has targeted those it was meant for, family caregivers of patients in hospice care and therefore close to death. Our reported 45% attrition rate was because the carers could not complete the post-intervention outcome measures due to patient deaths and not because they did not have time or did not want to do the intervention. In fact the qualitative feedback obtained from the 233 family carers in the intervention group was overwhelmingly positive [34]. Just a few stated that they would have preferred going through the CSNAT earlier to prepare them more for what is to come and some thought it was too early for them to know about the challenges they are going to face. The timing of the intervention when used in routine practice (without the constraints of the research process) is a call the nurses will need to make and gauge the best time to introduce the CSNAT.

### Limitations and direction for future research

Primary limitations of the study include the high attrition rate, unbalanced groups, and the non-randomised design being typical challenges reported in undertaking research in palliative care [35]. As explained below, these challenges operated at three levels: the individual clinician level, the structural or organisational level, and the research trial level.

We had aimed to recruit 440 participants (220 per condition) at baseline, which a priori sample size calculations indicated would be sufficient to adequately power the trial, with an anticipated 30% attrition rate. However, the observed attrition rate (45%) in the trial was greater than we initially estimated, due to a high rate of patient deaths over the study period. The trial was thus ultimately underpowered with a final sample size of 322 completers. Attrition rates as high as 69%, causing studies to be underpowered to detect intervention effects, were reported in a meta-analysis of several studies [32].

Use of a study completers sample may also impact on interpretation of findings. Given that the leading cause of attrition was patient deaths, drop outs are likely to have experienced increased strain and distress and caregiver burden over the study period. Limiting our analyses to only study completers may thus have overstated the effect of the intervention. However, we felt it was inappropriate to use estimation methods to replace missing data for drop-outs given the high rate of attrition and non-random nature of missing values.

Moreover, most if not all of the drop outs (other than those due to patient deaths, Fig 2) were not related to the intervention to warrant adopting a proposed approach that sits between intention-to-treat and per protocol analyses, which is the palliative-modified ITT analysis [36]. This new approach recommends including in the analysis those drop outs related to the intervention. However, the most common reasons for drop outs in our study were the progression of the disease or the patient reaching terminal stage. Another reason for dropping out, discharge from the service, was the result of the service operational changes whereby clients deemed not close to death were discharged and then readmitted at a later date if their condition deteriorated. These changes were made due to increased demand on the service and limited resources and took effect after the first time period of recruitment for the control arm, and therefore would have affected more the intervention arm.

Another limitation of our study was unbalanced groups, with almost three times as many participants in the intervention (n = 233) than control group (n = 89). This was due to slow recruitment at the start of the trial, when all sites were recruiting for the control group. The service nearly doubled (to 44) the number of nurses recruiting for the intervention to allow for quicker recruitment in the timeframe of the extended data collection period.

Not all caregivers would have been approached to participate and therefore there could be a risk of systematic selection bias. While we cannot rule out that gatekeeping took place, we believe that in many cases the decision to include a caregiver in the study would have been most likely influenced by the workload of the nurse on the day and how time-pressured she/he felt fitting in a number of patients that need to be visited. Nurses voiced their concerns at our meetings about how time consuming and burdensome the research process was, issues well reported in the literature [37,38]: Consenting caregivers and then contacting the researcher to conduct the baseline measurements before being able to undertake the CSNAT which would be in a second visit. Moreover the majority of these nurses worked part-time or job-shared and handover issues were not straightforward. However the profiles of non-participants and participants did not differ.

The issue of unbalanced groups ultimately is an inherent limitation of the stepped-wedge design; however, GLMM is generally robust to unbalanced groups. Relatedly, the intervention and control groups were found significantly different on a number of baseline characteristics. While analyses were adjusted for these differences, other potential underlying biases cannot be completely ruled out.

Finally, randomisation of intervention start times was not implemented in the present study due to logistic constraints. The service base that participated in the pilot study was assigned to start intervention first in the main trial in order to allow the remaining two bases a longer time to settle into this research study before they were ready to implement the CSNAT. Therefore, readiness to participate and experience in CSNAT may present as confounders, although it is encouraging to note that the Time x Base interaction effect was not significant in the GLMMs for all six outcomes, indicating that change in outcomes did not differ significantly between bases.

## Conclusion

Despite the described unavoidable challenges which operated at three levels, the individual clinician level, the structural or organisational level, and the research trial level, this study has provided sound evidence to support everyday clinical practice. The CSNAT implementation led to an improvement in caregiver strain during the caregiving period within this research context. While more research is needed on the effectiveness of the CSNAT in similar and other settings, the comprehensive quantitative and qualitative feedback obtained from all 233 caregivers [34] in the intervention group and the 44 nurses who implemented the intervention (under review), has been positive and encouraging that its implementation into routine practice can be explored in this service context.

As very few of such evidence-based interventions have been translated for or implemented in clinical practice settings, Northouse et al called for more collaborative effort between researchers and clinicians to move efficacy studies (phase III) to effectiveness studies (phase IV)[32]. Only then, we may see a reduction in publications reporting negatively on family caregiving experience, nationally and internationally.

Effective implementation of an evidence-informed tool represents a necessary step towards helping palliative care providers better assess and address caregiver needs, ensuring adequate family caregiver support and reduction in caregiver strain throughout the caregiving journey.
